# Laparoscopic treatment for an inflammatory fibroid polyp complicated with ileocecal intussusception: a case report and literature review

**DOI:** 10.1097/RC9.0000000000000238

**Published:** 2026-02-11

**Authors:** Hazem Beji, Jasser Rchidi, Houda Gazzah, Aymen Laaribi, Mahdi Bouassida, Hassen Touinsi

**Affiliations:** a Department of Gastrointestinal Surgery, Hospital Mohamed Taher Maamouri, Nabeul, Tunisia; b Faculty of Medicine of Tunis, University of Tunis El Manar, Tunis, Tunisia

**Keywords:** case report, ileocecal resection, inflammatory fibroid polyp, intussusception, laparoscopy

## Abstract

**Introduction and importance:**

Adult intussusception is a rare clinical entity, accounting for less than 5% of all cases and often associated with an underlying lesion. Inflammatory fibroid polyps (IFPs) are rare benign mesenchymal tumors that may act as a lead point for intussusception, most frequently in the small intestine. Herein, we report the case of a 52-year-old woman presenting with ileocecal intussusception secondary to an IFP.

**Case presentation:**

A 52-year-old woman presented with intermittent abdominal pain and symptoms of subacute obstruction. Imaging revealed an ileocolic intussusception due to a polypoid ileal lesion. Laparoscopic ileocecal resection was performed with primary ileocolic anastomosis. Histopathological analysis confirmed the lesion as an inflammatory fibroid lesion. The postoperative course was uneventful, corresponding to Clavien–Dindo Grade 0. The patient remained symptom-free at 6 months of follow-up.

**Clinical discussion:**

IFPs most often arise in the stomach and small intestine. In the small bowel, IFPs can serve as a lead point for intussusception, but ileocecal presentations remain uncommon. The laparoscopic approach is safe and provides excellent visualization and is associated with reduced pain and faster recovery. Complete resection of IFPs is curative with rare recurrence rates. Definitive diagnosis relies on histopathology and immunohistochemistry.

**Conclusion:**

This case highlights the diagnostic challenges of adult intussusception, particularly when imaging is inconclusive. IFPs should be considered as a differential diagnosis. Moreover, it underscores the importance of laparoscopic surgical management both for diagnostic and therapeutic purposes.

## Introduction

Adult intussusception is an uncommon cause of intestinal obstruction, representing approximately 1%–5% of cases^[1]^. In contrast to pediatric cases, adult intussusception is usually secondary to a pathological lead point that may be benign or malignant^[2]^. Inflammatory fibroid polyps (IFPs), or Vanek’s tumors, are rare benign mesenchymal lesions most often found in the stomach and small intestine^[3]^. IFPs presenting at the ileocecal junction and acting as a lead point are exceptionally uncommon^[4]^. Herein, we report the case of a 52-year-old woman presenting with ileocecal intussusception secondary to an IFP.

**HIGHLIGHTS**

- This report describes a rare case of ileocecal intussusception in an adult caused by an inflammatory fibroid polyp.
- It demonstrates the feasibility of laparoscopic ileocecal resection despite bowel obstruction.
- It highlights the role of CD34+/DOG1− immunohistochemistry in confirming diagnosis.
- It illustrates that timely laparoscopic surgery provides both diagnosis and cure in uncertain adult intussusception.

This work has been reported in line with the Surgical CAse REport (SCARE) 2025 criteria^[5]^.

## Presentation of a case

A 52-year-old woman with no significant medical or surgical history experienced a 2-month history of paroxysmal, crampy abdominal pain predominantly in the right lower quadrant, accompanied by occasional nausea, intermittent vomiting, and reduced oral intake. The pain escalated in intensity, becoming severe, leading to presentation to the emergency department. The patient was a non-smoker, did not consume alcohol, and had no relevant family history of gastrointestinal disease. The patient reported no weight loss, fever, or alteration in bowel habits.

Examination showed mild abdominal distension and right lower-quadrant tenderness without peritoneal signs; no mass was palpable. Bowel sounds were hyperactive.

**Figure 1.**
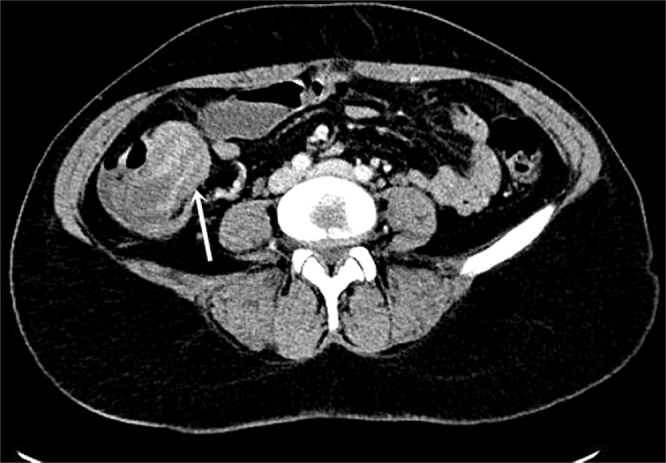
Axial contrast-enhanced CT image demonstrating the classic “target sign” (white arrow) of an ileocecal intussusception, which prompted urgent surgical exploration.

Urgent laboratory tests, including complete blood count, electrolytes, renal function, and C-reactive protein, were within normal limits. A contrast-enhanced computed tomography (CT) scan revealed a characteristic “target” or “bowel-within-bowel” appearance of an ileocecal intussusception with upstream small-bowel dilatation and air–fluid levels. A well-defined polypoid ileal lesion was identified (Fig. 1).

There was no delay between the patient’s presentation and her diagnosis and management. The patient was urgently admitted to our surgery department. She was kept *nil per os*, and preoperative preparation included establishment of intravenous access with continuous monitoring, placement of a nasogastric tube, pre-anesthetic assessment, and a detailed explanation of the planned procedure.

**Figure 2.**
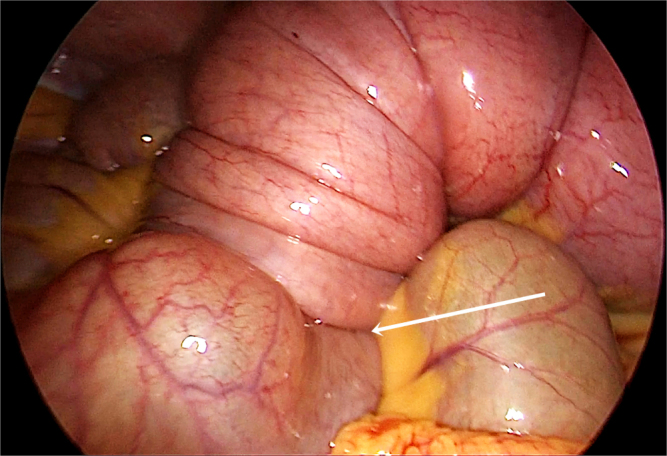
Intraoperative images demonstrating the intussusception (arrow), highlighting the extent of bowel telescoping and confirming the preoperative radiological findings.

**Figure 3.**
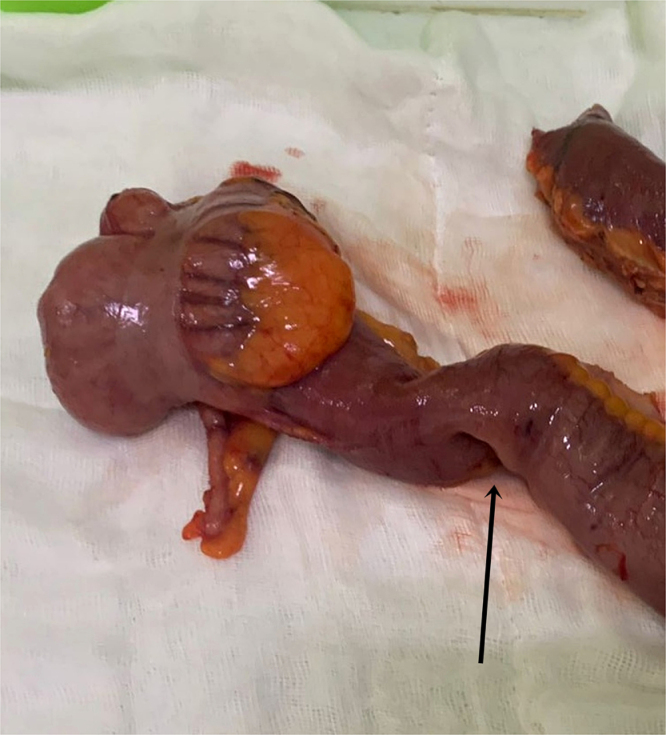
Gross examination of the resected surgical specimen revealed an ileal inflammatory fibroid polyp (arrow), identified as the pathological lead point responsible for the intussusception.

Given these findings, laparoscopic surgery was undertaken the following day by an experienced senior surgeon. On exploration, we found the ileocecal intussusception (Fig. 2). The terminal ileum and right colon were exposed, and a retro-mesenteric “tent” was created by a careful medial-to-lateral, inferior-to-superior dissection. Direct visualization of the duodenum was achieved early. The medial dissection was stopped after visualization of the gallbladder. Lateral attachments were then sectioned, and the right colon was completely mobilized. The mesentery was divided proximal to the bowel, knowing that the lesion was not suspicious on the imaging. The specimen was delivered through a small right transverse incision (Fig. 3). An extracorporeal hand-sewn two-layer, side-to-side ileocolic anastomosis was constructed.

The postoperative course was unremarkable, with no complications recorded, corresponding to a Clavien–Dindo Grade 0 outcome. Oral intake resumed on postoperative day 2, and the patient was discharged on day 4. Histopathology showed a submucosal spindle-cell lesion with prominent eosinophilic infiltration and concentric perivascular “onion-skin” fibrosis. Immunohistochemistry was positive for CD34 and negative for DOG1, S100, and c-KIT, consistent with an IFP. No malignant features were identified. Throughout the follow-up period, at 1, 3, and 6 months, the patient remained asymptomatic. During her follow-up visits, she expressed satisfaction with the minimally invasive approach and rapid recovery, reporting complete resolution of all preoperative symptoms.

## Clinical discussion

This case demonstrates a successful laparoscopic treatment for an ileal IFP revealed by ileocolic intussusception.

Adult intussusception differs from pediatric disease in etiology, presentation, and management, with most adult cases driven by a definable lead point^[6]^.

IFPs are rare benign mesenchymal tumors of the gastrointestinal tract, most often arising in the stomach and small bowel^[3]^. It was first described by Vanek in 1949^[7]^.

Vague, intermittent symptoms, typically crampy abdominal pain with subacute obstructive episodes, commonly delay diagnosis^[8]^.

In the small bowel, IFPs can serve as a lead point for intussusception, but ileocecal presentations remain uncommon^[9]^. Recent case-based literature continues to underscore their rarity as a cause of adult intussusception^[10]^.

Differential diagnoses are essentially gastrointestinal stromal tumors and leiomyomas^[11,12]^.

In adults, the majority of intussusceptions are associated with an underlying malignancy, making a benign lead point, such as an IFP, unexpected and difficult to anticipate preoperatively^[9]^. Moreover, IFPs are frequently small, submucosal, and radiologically subtle, which limits their detectability on CT imaging. These factors contribute to diagnostic uncertainty and reinforce the need for a high index of suspicion and timely surgical exploration when symptoms persist^[3]^.

Contrast-enhanced CT scan is the diagnostic modality of choice in adults, typically demonstrating a “target” or “sausage” sign and mesenteric fat/vascular invagination^[13]^. Nevertheless, small submucosal lesions may be radiologically occult, necessitating clinical correlation and operative exploration when symptoms persist or obstruction is present^[8]^. Colonoscopy has limited yield for distal ileal pathology in this context, and a normal examination does not exclude a submucosal lead point^[2]^.

Surgical resection is recommended for adult intussusception due to the risk of ischemia or malignancy^[3]^.

A laparoscopic approach provides excellent visualization, facilitates en-bloc resection when indicated, and is associated with reduced pain and faster recovery in appropriately selected patients^[14]^. The laparoscopic approach can be difficult due to the distension of the small bowel caused by intussusception. In the presence of operative difficulties, conversion to laparotomy should not be delayed to avoid bowel injuries^[15,16]^.

Definitive diagnosis relies on histopathology and immunohistochemistry. Complete excision of IFPs is curative in the vast majority of cases, with rare recurrence^[9]^. In immunohistochemistry, IFPs are characteristically CD34-positive and DOG1/c-KIT-negative, aiding distinction from gastrointestinal stromal tumors^[17]^. Histologically, they comprise spindle-cell proliferation within a fibromyxoid stroma with prominent eosinophils and perivascular concentric fibrosis.

This case report provides detailed clinical, radiological, surgical, and histopathological documentation of a rare adult ileocecal intussusception caused by an IFP.

Its main strength lies in the rarity of such cases reported in the literature, particularly those managed successfully via a laparoscopic approach. The case illustrates the importance of maintaining a broad differential diagnosis in adult intussusception, recognizing subtle imaging findings, and tailoring the surgical approach to the patient’s condition, including bowel distension and the severity of obstruction.

Limitations include the single-patient, single-center design, which limits generalizability, and the relatively short follow-up of 6 months. The patient was treated in a tertiary care center with laparoscopic expertise; results may not translate to centers with limited minimally invasive experience.

Despite these limitations, the report contributes meaningful clinical insight into the diagnosis and laparoscopic management of this uncommon entity.

## Conclusion

Adult ileocecal intussusception caused by an IFP is rare and diagnostically challenging. When imaging suggests intussusception, a timely laparoscopic approach provides both diagnosis and cure, with the benefits of minimally invasive surgery, while allowing the surgical management to be tailored to the patient’s condition.

## Data Availability

Not applicable.

## References

[1] Azar T Berger DL. Adult intussusception. Ann Surg 1997;226:134–38. 9296505 10.1097/00000658-199708000-00003 PMC1190946

[2] T Chand J Rakesh R Ganesh MS. Adult intussusception: a systematic review of current literature. Langenbeck’s archives of surgery 2024;409:235. 10.1007/s00423-024-03429-2 39085533

[3] Nonose R Valenciano JS Silva CM. Ileal intussusception caused by Vanek’s tumor: a case report. Case Rep Gastroenterol 2011;5:110–16. 21503167 10.1159/000326930 PMC3078240

[4] Wysocki AP Taylor G Windsor JA. Inflammatory fibroid polyps of the duodenum: a review of the literature. Dig Surg 2007;24:162–68. 17476106 10.1159/000102099

[5] Kerwan A Al-Jabir A Mathew G. Revised Surgical CAse REport (SCARE) guideline: An update for the age of Artificial Intelligence. Premier Journal of Science 2025;10:100079.

[6] Kumar K Noori MR Patel KM. Rare diagnosis of intestinal lipomatosis complicated by intussusception in an adult: A case report. Int J Surg Case Rep 2017;39:339–42. 28898799 10.1016/j.ijscr.2017.08.038 PMC5597814

[7] Vanek J. Gastric submucosal granuloma with eosinophilic infiltration. Am J Pathol 1949;25:397–411. 18127133 PMC1942901

[8] Aref H Nawawi A Altaf A. Transient small bowel intussusception in an adult: case report with intraoperative video and literature review. BMC Surg 2015;15:36. 25881028 10.1186/s12893-015-0020-6 PMC4416349

[9] Bratke G Nowicki M Michalak M. Inflammatory Fibroid Polyp Causing Ileal Intussusception in an Adult. Medicina (B Aires) 2022;58:310.

[10] Wang Y Wu Y Chen J. Rare invasive inflammatory fibroid polyp causing intussusception in an adult: a case report. Medicine (Baltimore) 2025;104:e31220. 10.1097/MD.0000000000041956 PMC11957626 40163344

[11] Beji H Bouassida M Kallel Y. Leiomyoma of the esophagus: A case report and review of the literature. Int J Surg Case Rep 2022;94:107078. 35439728 10.1016/j.ijscr.2022.107078 PMC9027349

[12] Li H Ren H Sun H. Jejunojejunal intussusception with chronic bleeding caused by gastrointestinal stromal tumor: a case report and literature review. J Gastrointest Oncol 2022;13:1481–88. 35837170 10.21037/jgo-22-301 PMC9274023

[13] Tormane MA Laamiri G Beji H. Primary mantle-cell lymphoma of small intestine presenting with intussusceptions: A case report and review of the literature. Int J Surg Case Rep 2024;121:109963. 38954973 10.1016/j.ijscr.2024.109963 PMC11263630

[14] Belhamidi MS Aitali A Tarchouli M. Ileo-ileal intussusception in adult caused by Vanek’s tumor: a report of five cases. Int Surg J 2015;2:595–98.

[15] Takeuchi K Tsuzuki Y Ando T. The diagnosis and treatment of adult intussusception. J Clin Gastroenterol 2003;36:18–21. 12488701 10.1097/00004836-200301000-00007

[16] Goh BK Quah HM Chow PK. Predictive factors of malignancy in adult intestinal intussusception. World J Surg 2006;30:1300–04. 16773257 10.1007/s00268-005-0491-1

[17] Bassam A. Vanek’s tumor of the small bowel in adults. World Journal of Gastroenterology 2012;21:4802–4808. 10.3748/wjg.v21.i16.4802 PMC4408452 25944993

